# Valorization of the Invasive Gastropod *Rapana venosa* Through Cold Marination and Vacuum Packaging: Quality Characteristics and Shelf‐Life Assessment Under Refrigerated Storage

**DOI:** 10.1002/fsn3.72104

**Published:** 2026-07-10

**Authors:** Bilge Bilgin Fıçıcılar, Koray Korkmaz

**Affiliations:** ^1^ Department of Fisheries Technologies Engineering Fatsa Marine Sciences Faculty, Ordu University Fatsa Ordu Turkey

**Keywords:** cold marination, marination, *Rapana venosa*, Rapana whelk, ready‐to‐eat aquatic product

## Abstract

*Rapana venosa*, an ecologically invasive yet commercially valuable gastropod species, represents a promising raw material for value‐added seafood products. In this study, boiled *R. venosa* meat was cold‐marinated using two formulations (3.8% acetic acid + 5% salt and 3.9% acetic acid + 8% salt), vacuum‐packaged with sunflower oil, and stored at 4°C for 11 months. Total viable counts, psychrotrophic bacteria, and coliforms remained below the detection limit (< 10 cfu/g) throughout storage. pH increased significantly from 3.88 to 4.92 (A) and 3.76 to 4.45 (B) (*p* < 0.05). TVB‐N rose to 19.43 (A) and 17.69 (B) mg N/100 g, well below the 30–35 mg N/100 g spoilage threshold, with Sample B accumulating significantly less (*p* < 0.05). Free fatty acids reached 14.11% (A) and 16.27% (B) and TBA reached 1.65 and 1.76 mg MDA/kg, whereas peroxide value stayed low and stable (0.41–0.62 meq O_2_/kg). Sensory acceptability declined but stayed above the threshold (3.50 for A, 3.25 for B at Month 11; *p* < 0.05). Marination markedly altered the fatty acid profile: raw meat was rich in EPA (6.07%) and DHA (19.26%), whereas marinated samples were dominated by linoleic (~56%) and oleic (~31%) acids, with n‐3 PUFAs reduced to ~0.3%, reflecting dilution by sunflower oil rather than oxidative degradation. Overall, cold marination with vacuum packaging and oil preservation ensured microbial safety, maintained acceptable sensory quality, and extended refrigerated shelf life to 11 months, supporting valorization of *R. venosa* as a ready‐to‐eat product.

## Graphical abstract

Processing scheme of Rapana whelk (*Rapana venosa*) into a ready‐to‐eat marinated product through cold marination, sunflower oil addition, vacuum packaging, and 11 months of refrigerated storage (4°C), followed by evaluation of microbiological, chemical, sensory, and shelf‐life characteristics.

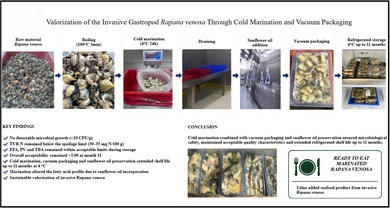

## 1 Introduction

The veined rapa whelk, *Rapana venosa*, is a marine gastropod mollusk belonging to the family Muricidae and is native to the western Pacific region, including the Sea of Japan, the Yellow Sea, the East China Sea, and the Bohai Sea. Over recent decades, this species has become invasive in several regions worldwide, notably the Black Sea, the Adriatic and Aegean Seas, and the Chesapeake Bay (Aydın et al. 2016; Mann and Harding 2000; Zolotarev 1996). As a highly successful invader, *R. venosa* spread to the coastal waters of Bulgaria, Türkiye, and Romania between 1959 and 1972 (Marmov 1990). Its introduction to these areas is widely attributed to the unintentional transport of larvae or juveniles during oyster translocations from the Sea of Japan for aquaculture enhancement (Drapkin 1963; Zolotarev 1996). *R. venosa* is a predatory species that primarily feeds on bivalves, particularly mussels, and exhibits a spawning period between June and early August (Sağlam et al. 2009).

Despite the negative ecological impacts in invaded ecosystems, it has gained significant economic importance owing to its high commercial value in international seafood markets. Demand is particularly strong in East Asia, where the species is widely traded and consumed, especially in China, South Korea, and Japan (Yuan 1992). At the same time, Spain is an important destination market in Europe. Although native populations in the western Pacific support local consumption, global demand is increasingly supplied by imports from non‐native regions, most notably the Black Sea (Mann and Harding 2000). This trade linkage is clearly reflected in Türkiye's export statistics. According to data from the Eastern Black Sea Exporters' Association (DKİB), 1017 t of rapa whelk were exported from Türkiye to eight countries during the January–September 2025 period, generating a total revenue of USD 10.68 million. South Korea, China, and Spain were the leading destination markets, with export revenues of USD 5.26 million, USD 1.61 million, and USD 1.31 million, respectively, underscoring the dominant role of East Asian demand alongside emerging European markets in shaping the international trade of *R. venosa* from the Black Sea region of Türkiye.

In recent years, changing consumer lifestyles and preferences have led to increased demand for ready‐to‐eat and minimally processed seafood products that offer convenience, extended shelf life, and high sensory quality. In this context, the development of value‐added products derived from *R. venosa* represents a practical approach for improving its market utilization while supporting the sustainable use of this species. Marination is a well‐established semi‐preservation method for fish and seafood, based on the treatment of muscle tissues with marinade solutions containing salt and organic acids, often supplemented with sugar, spices, oils, or natural acid sources such as vinegar, fruit juices, or wine. Marinade solutions are widely used to enhance sensory attributes, including tenderness, juiciness, flavor, and aroma, while simultaneously extending product shelf life (Cadun et al. 2005). The preservative effect of marination is primarily associated with reduced pH, lowered water activity, and the inhibitory action of salt and acids on microbial growth and enzymatic activity, with these effects generally increasing with marinade concentration and marinating time (Duyar and Eke 2009; Yashoda et al. 2005). *R. venosa* has considerable commercial importance, and its utilization in international seafood markets has increased substantially in recent years. Although numerous studies have focused on its ecology, fishery, reproductive biology, and nutritional composition, information regarding the development of value‐added ready‐to‐eat products and their long‐term storage stability remains limited. In particular, studies evaluating the effects of cold marination and prolonged refrigerated storage on the quality and shelf life of *R. venosa* are scarce.

Therefore, the aim of this study was to evaluate the suitability of *R. venosa* meat for producing a cold‐marinated, ready‐to‐eat product and to determine its shelf life during refrigerated storage. To achieve this objective, two marination formulations differing in salt and acetic acid concentrations were compared for microbiological safety, physicochemical characteristics, sensory properties, color parameters, and fatty acid composition over an 11‐month storage period at 4°C. The findings of this study provide new information on the processing and preservation of *R. venosa* and contribute to the development of value‐added products from this economically important invasive species.

## 2 Materials and Methods

### 2.1 Materials

Fresh *R. venosa* meat was obtained from Nam Su Ürünleri (Fatsa, Ordu, Türkiye). The rapa whelks were harvested from the Black Sea and processed at the facility prior to purchase. The specimens were cleaned and removed from their shells, and no freezing or chlorination was applied prior to further processing. The preliminary processing steps performed at the facility included cooking in water at 100°C for 3 min, washing, removal of shell fragments, meat washing, grading, and weighing. Individual rapa whelk meats weighed approximately 20–40 g. A total of 7.0 kg of rapa whelk meat was used in the study, with 3.5 kg allocated to each marination treatment. Following these processing steps, the material was transported under refrigerated conditions to Sastaş A.Ş. (Samsun, Türkiye), where marination, vacuum packaging, storage, and subsequent analyses were carried out.

### 2.2 Marination Procedure

Two marinated samples were prepared and coded as Sample A and Sample B. For each treatment, 3.5 kg of *rapana* meat was immersed in 5.0 kg of marination solution (rapana:marination solution was used as 7:10 [w/v]) and marinated for 24 h under refrigerated conditions.

Sample A: Marination solution adjusted to 3.8% acidity (as acetic acid) (Ajinomoto—Kükre gıda) and 5% salt (Andaçlar).

Sample B: Marination solution adjusted to 3.9% acidity (as acetic acid) (Ajinomoto—Kükre gıda) and 8% salt (Andaçlar). The marination conditions used in the study were selected based on preliminary trial studies conducted prior to the main experiment to obtain products with acceptable sensory characteristics and technological suitability.

**FIGURE 1:**
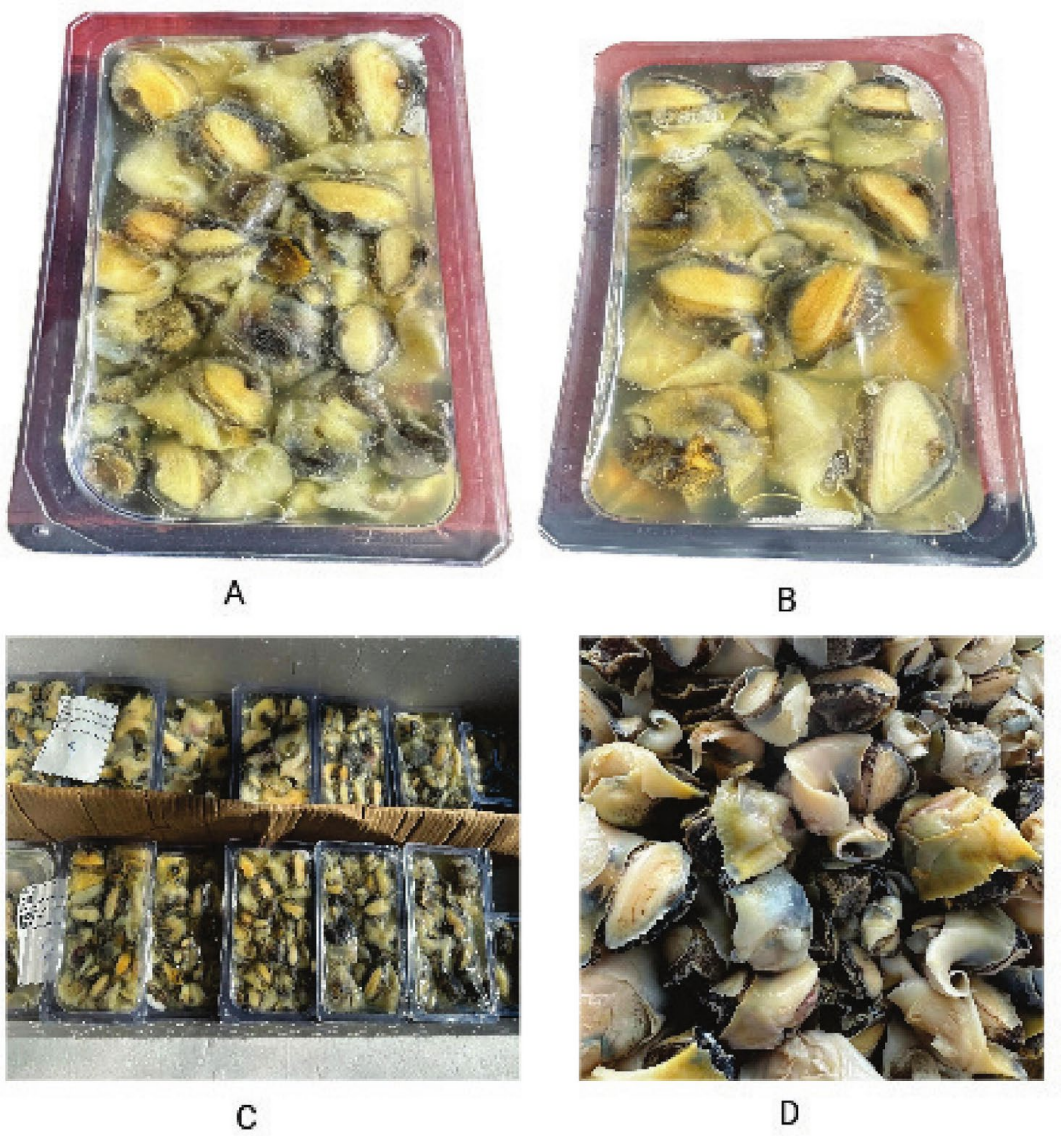
Visual appearance of marinated *Rapana venosa* samples and raw material. (A) Sample A after marination and packaging. (B) Sample B after marination and packaging. (C) Overview of all packaged marinated samples (A and B groups). (D) Raw *Rapana venosa* meat prior to processing.

After marination, sunflower oil was added, and the samples were vacuum‐packaged in plastic packages. The photograph of the raw Rapana meat and marinated *R. venosa* is given in Figure 1. All packages were stored under refrigerated conditions (4°C) for a period of 11 months. Sensory, chemical, and microbiological analyses were conducted at 0, 3, 8, and 11 months of storage. At each sampling interval, three independent vacuum‐packaged samples were randomly selected and opened for analysis. Microbiological analyses were performed immediately after opening the packages, followed by the remaining analyses. Before analysis, the samples were homogenized. Each package was used only once, and all data were obtained from three independent packages at each sampling point.

### 2.3 Methods

#### 2.3.1 pH

Samples (10 g) were homogenized with 10 mL of distilled water, and measurements were conducted in triplicate (Bongiorno et al. 2018).

#### 2.3.2 Peroxide Value (PV)

Peroxide value was assessed using the iodometric titration technique, and the results were reported as milliequivalents (meq) of active oxygen per kilogram of oil (AOAC 2000).

#### 2.3.3 Thiobarbituric Acid (TBA) Value

The extent of secondary lipid oxidation was evaluated by determining malondialdehyde (MDA) equivalents spectrophotometrically at a wavelength of 532 nm, following the method of Tarladgis et al. (1960).

#### 2.3.4 Free Fatty Acids (FFA)

Free fatty acid levels were measured by titration with 0.1 N NaOH in the presence of phenolphthalein indicator and expressed as a percentage of oleic acid (AOCS 1997).

#### 2.3.5 Total Volatile Basic Nitrogen (TVB‐N)

TVB‐N was quantified following the method of Antonacopoulos and Vyncke (1989), and the results were expressed as mg TVB‐N per 100 g of muscle.

#### 2.3.6 Microbiological Analysis

Total psychrotrophic aerobic counts (TPAC) and total mesophilic aerobic counts (TMAC) were determined using Plate Count Agar (PCA; Merck) by the pour‐plate method (ISO 2013). Plates were incubated at 4°C for 14 days (TPAC) and at 30°C for 24–48 h (TMAC). Coliform bacteria were enumerated on Violet Red Bile Agar (VRBA; Merck) after incubation at 35°C for 18–24 h (ISO 2006). Colonies were counted, and results were expressed as log CFU/g.

#### 2.3.7 Sensory Analysis

Sensory evaluation was done at 0, 3, 8, and 11 months of refrigerated storage for Sample A and Sample B. Panelists were selected from individuals familiar with seafood products and were informed about the product and evaluation procedure before participation. Written informed consent was obtained from all panelists. Color, brightness, appearance, odor, greasiness, flavor, texture, and overall acceptability were assessed using a 9‐point hedonic scale (1 = dislike extremely; 9 = like extremely). Samples were coded with random three‐digit numbers and presented under controlled conditions. Water was provided between samples.

#### 2.3.8 Color Measurements

Color measurements of the marinades were performed in triplicate using a Konica Minolta CM‐5 colorimeter (Osaka, Japan), and L*, a*, and b* values were recorded.

#### 2.3.9 Fatty Acid Composition

Fatty acid methyl esters (FAMEs) were prepared by acid‐catalyzed transesterification using 2% H_2_SO_4_ in methanol and analyzed by gas chromatography with flame ionization detection (GC‐FID; Agilent Technologies, Santa Clara, CA, USA). Separations were performed using a capillary column (TR‐CN100, 100 m × 0.25 mm i.d., 0.20 μm film thickness; TEKNOKROMA, Barcelona, Spain). The injector and detector temperatures were set at 250°C. The oven temperature program started at 140°C (held for 5 min), increased to 240°C at a rate of 4°C/min, and was held at 240°C for 20 min. Helium was used as the carrier gas at a constant flow rate of 1 mL/min. Samples (1 μL) were injected in split mode with a split ratio of 100:1. Fatty acids were identified by comparing their retention times with those of a certified FAME standard mixture (Supelco 37 Component FAME Mix; Sigma‐Aldrich, St. Louis, MO, USA), and results were expressed as percentage of total identified fatty acids.

### 2.4 Statistical Analysis

Statistical analysis was performed using IBM SPSS Statistics 31.0 (IBM Corp., Armonk, NY, USA). All experimental data were expressed as mean ± standard deviation (*p* < 0.05). To evaluate the effects of different marination formulations and storage duration (0, 3, 8, and 11 months) on the physicochemical, chemical, and sensory parameters of marinated *R. venosa*, one‐way analysis of variance (ANOVA) was conducted. Following ANOVA, Tukey's multiple comparison test was applied to determine significant differences between means at a significance level of (*p* < 0.05). In the presentation of results, different superscript lowercase letters are used to denote significant changes during storage within the same formulation, whereas uppercase letters indicate significant differences between the two formulations at the same sampling point.

## 3 Results and Discussion

### 3.1 Chemical Analysis

#### 3.1.1 pH

The initial pH values were 3.88 for Sample A and 3.76 ± 0.01 for Sample B (Table 1). These acidic values reflect the effect of acetic acid‐based marination, which is widely reported to reduce pH and improve microbial stability in seafood products (Goulas and Kontominas 2007; Huss 1995). The slightly lower initial pH in Sample B is consistent with its higher acetic acid and salt concentration, suggesting a stronger acidification effect.

**TABLE 1:** Changes in physicochemical and lipid oxidation parameters of marinated *Rapana venosa* samples (A and B) during refrigerated storage.

Storage	Month 0		Month 3		Month 8		Month 11	
Sample	A	B	A	B	A	B	A	B
pH	3.88 (0.01^aB^)	3.76 (0.01^aB^)	3.85 (0.01^aA^)	3.88 (0.01^aB^)	4.48 (0.11^bA^)	4.24 (0.05^bB^)	4.92 (0.06^cA^)	4.45 (0.22^cB^)
Free fatty acids (%)	0.54 (0.04^aA^)	4.85 (0.07^aB^)	6.94 (0.13^bA^)	9.50 (0.57^bB^)	12.20 (0.11^cA^)	14.58 (0.42^cB^)	14.11 (0.89^dA^)	16.27 (0.34^dA^)
PV (Meq O_2_/kg)	0.60 (0.04^aA^)	0.41 (0.04^aA^)	0.59 (0.04^aA^)	0.57 (0.06^aA^)	0.62 (0.08^aA^)	0.60 (0.03^aA^)	0.55 (0.20^aA^)	0.57 (0.12^aA^)
TBA (mg MDA/kg)	0.16 (0.04^aA^)	0.17 (0.03^aA^)	0.46 (0.04^bA^)	0.60 (0.12^bA^)	1.21 (0.02^cA^)	1.31 (0.04^cA^)	1.65 (0.03^dA^)	1.76 (0.06^dA^)
TVB‐N (mg N/100 g)	6.50 (0.46^aA^)	6.21 (0.10^aA^)	11.30 (0.31^bA^)	10.25 (0.15^bA^)	16.62 (0.40^cA^)	14.57 (0.48^cB^)	19.43 (0.69^dA^)	17.69 (0.85^dB^)

*Note:* Values are expressed as mean ± standard deviation (*n* = 3). Different lowercase letters indicate significant differences among storage times within the same formulation, whereas different uppercase letters indicate significant differences between formulations at the same storage time (*p* < 0.05). Sample A: 3.8% acetic acid, 5% salt. Sample B: 3.9% acetic acid, 8% salt.

Within each formulation, pH values increased significantly during storage (*p* < 0.05). In Sample A, pH rose from 3.88 at Month 0 to 4.92 at Month 11, while in Sample B it increased from 3.76 to 4.45 over the same period (Table 1). Similar increases in pH during refrigerated storage of marinated molluscan products have been attributed to the formation of alkaline nitrogenous compounds such as ammonia and other volatile bases resulting from enzymatic and microbial protein degradation (Huss 1995). The concurrent rise in TVB‐N values observed in the present study supports this explanation. Significant differences between formulations were observed at Months 8 and 11, with Sample B maintaining lower pH values (*p* < 0.05). This may be attributed to its higher acid–salt concentration, which likely enhanced preservation efficiency. Similar results have been reported in marinated mussels and marinated gastropods such as *Hexaplex trunculus* and *Bolinus brandaris* during refrigerated storage, where increased acid levels were associated with lower pH and improved storage stability (Aveiro et al. 2007; Yavuz 2012).

Although *R. venosa* meat is a low‐fat raw material (≈0.5% lipid) (Popova et al. 2017), FFA values increased significantly during storage (Table 1). The progressive increase in free fatty acids during storage may result from hydrolytic reactions of marine lipids. In addition, the sunflower oil used as the covering medium may have contributed to the increase in FFA due to its own susceptibility to hydrolysis during prolonged storage. Lipid hydrolysis is influenced by long contact with the oil phase, salt concentration, and storage time, particularly in PUFA‐rich matrices such as sunflower oil (Bilgin Fıçıcılar et al. 2018; Shahidi and Zhong 2010).

Peroxide value (PV) serves as an indicator of primary lipid oxidation by measuring hydroperoxide accumulation, whereas thiobarbituric acid (TBA) values reflect the formation of secondary oxidation products, such as malondialdehyde (MDA) (Shahidi and Zhong 2005; O'Keefe and Pike 2010). In this study, PV remained relatively low and stable throughout the storage period (0.41–0.62 meq O_2_/kg), whereas TBA values exhibited a progressive increase (Table 1). No significant differences were observed between formulations for PV values (*p* > 0.05). However, TBA values were significantly higher in sample group B at advanced storage periods (*p* < 0.05), suggesting that secondary oxidation processes were more influenced by formulation differences than primary peroxide formation.

The stability of PV suggests that primary oxidation products did not accumulate significantly. However, since lipid hydroperoxides are unstable, they often undergo rapid decomposition into secondary volatile compounds, including aldehydes and ketones, particularly during extended storage. Therefore, the observed rise in TBA values across both sample groups (A and B) can be attributed to the degradation of transient hydroperoxides into more stable secondary metabolites rather than the sustained accumulation of primary products (Choe and Min 2006).

Total volatile basic nitrogen (TVB‐N) is a critical indicator of spoilage in seafood, reflecting the accumulation of ammonia, TMA, and other volatile amines resulting from bacterial activity and endogenous enzyme action (Giménez et al. 2002; Huss 1995). In this study, TVB‐N values increased significantly in both samples throughout the 11‐month storage period at 4°C (*p* < 0.05), rising from an initial average of 6.35 mg N/100 g to 19.43 and 17.69 mg N/100 g for Sample A and B, respectively (Table 1). Despite this progressive increase, all values remained well below the generally accepted spoilage threshold of 30–35 mg N/100 g for fishery products, suggesting that both formulations effectively maintained freshness during long‐term cold storage (Commission Regulation [EC] 2005; Özogul et al. 2004).

The fact that microbial loads (including TVC, psychrotrophic bacteria, and coliforms) remained undetectable or negligible throughout the storage period confirms that the observed chemical changes were not bacterial‐mediated. Instead, the gradual rise in TVB‐N and pH can be attributed to the slow, internal enzymatic degradation of proteins into volatile nitrogenous bases.

Notably, Sample B exhibited even lower TVB‐N accumulation than Sample A (*p* < 0.05). Since microbial activity was absent in both, this difference directly demonstrates that the higher salt and vinegar ratio in Sample B was more effective at denaturing or inhibiting the endogenous enzymes (such as cathepsins or calpains) within the *R. venosa* tissue. This synergistic effect of low pH and high osmotic pressure effectively maintained the muscle structure, preserving the chemical integrity of the meat even under long‐term refrigerated conditions.

### 3.2 Microbiological Assessment

Total viable counts (TVC), psychrotrophic bacteria, and total coliforms remained below detection limits (< 10 cfu/g) during 11 months of refrigerated storage in Sample A and Sample B groups. The suppressed microbial activity can be attributed to the combined preservative effects of low pH, increased salt levels, and the anaerobic conditions ensured by vacuum packaging and immersion in sunflower oil.

The absence of detectable total viable counts and psychrotrophic bacteria (< 10 cfu/g) in the current study is in agreement with the findings of Kilinc and Cakli (2005), who reported that these microbial populations were not detected in pasteurized sardine marinades throughout refrigerated storage. Similarly, these results align with the findings of Bilgin Fıçıcılar and Gençcelep (2020), who observed that marinated anchovies stored in sunflower oil at 4°C exhibited microbial loads below detection limits (10 cfu/g) for up to 7 months. The extension of this stability to 11 months in the present study further demonstrates the potency of the hurdle technology employed. As no microbial growth was detected throughout storage, alterations in TVB‐N, pH, and FFA can be attributed to endogenous enzymatic processes and non‐biological chemical reactions, excluding bacterial catabolism as a contributing factor.

### 3.3 Sensory Evaluation

The acceptability scores for color, brightness, appearance, odor, greasiness, flavor, texture, and overall acceptability are given in Table 2.

**TABLE 2:** Sensory evaluation scores of marinated *Rapana venosa* samples (A and B) during refrigerated storage.

Storage	Month 0		Month 3		Month 8		Month 11	
Sample	A	B	A	B	A	B	A	B
Color	4.86 (0.38^a^)	4.71 (0.49^a^)	4.29 (1.11^ab^)	4.29 (0.49^ab^)	3.75 (0.50^b^)	3.75 (1.26^b^)	2.75 (0.50^c^)	2.75 (0.50^c^)
Brightness	5.00 (0.00^a^)	4.86 (0.38^a^)	4.14 (1.21^ab^)	4.14 (0.90^ab^)	4.00 (0.00^b^)	4.25 (0.96^b^)	2.75 (0.50^c^)	3.00 (0.82^c^)
Appearance	4.29 (0.95^a^)	4.29 (0.95^a^)	4.43 (0.98^a^)	4.00 (1.00^a^)	4.00 (0.82^ab^)	4.25 (0.96^ab^)	3.75 (0.50^b^)	3.75 (0.50^b^)
Odor	4.71 (0.49^a^)	4.57 (0.53^a^)	4.00 (0.82^ab^)	3.29 (0.95^b^)	4.25 (0.96^ab^)	4.00 (1.41^ab^)	3.75 (0.50^b^)	3.75 (0.50^b^)
Greasiness	3.86 (0.69^a^)	3.86 (0.90^a^)	3.29 (1.50^ab^)	2.86 (1.07^b^)	3.75 (0.50^a^)	3.50 (0.58^a^)	3.25 (0.96^b^)	3.50 (1.00^b^)
Flavor	4.29 (0.76^a^)	4.43 (0.53^a^)	4.14 (0.90^a^)	2.86 (1.35^b^)	3.75 (0.50^ab^)	3.00 (1.41^b^)	3.25 (0.50^b^)	3.50 (0.58^b^)
Texture	4.00 (0.82^a^)	4.00 (0.82^a^)	3.86 (1.35^a^)	3.43 (1.13^a^)	4.00 (0.00^a^)	3.75 (0.50^a^)	3.25 (0.50^b^)	3.00 (0.82^b^)
Overall acceptability	4.71 (0.49^a^)	4.50 (0.50^a^)	3.79 (1.41^ab^)	2.64 (1.38^b^)	4.33 (0.41^a^)	3.75 (0.87^ab^)	3.50 (0.41^b^)	3.25 (0.87^b^)

*Note:* Values are expressed as mean ± standard deviation. Different lowercase letters within the same formulation indicate significant differences among storage times (*p* < 0.05). Sample A: 3.8% acetic acid, 5% salt. Sample B: 3.9% acetic acid, 8% salt.

The sensory profile of marinated *R. venosa* samples followed a gradual and predictable decline over the 11‐month storage period, while certain sensory attributes, particularly color at Month 11 and overall acceptability of Sample B at Month 3, fell below the acceptability threshold of 3.00, the products generally maintained acceptable sensory quality throughout storage, with overall acceptability scores exceeding 3.00 at the end of the storage period. Starting with high scores across all parameters (ranging from 3.86 to 5.00 at Month 0), both samples maintained their sensory appeal well into the later stages of storage.

#### 3.3.1 Color, Brightness, and Appearance

At the start of storage (Month 0), color and brightness scores were at their peak for both groups, reflecting the high initial quality of the samples. Then these scores showed a significant decrease toward the end of storage, reaching 2.75 and 3.00, respectively, by Month 11 (*p* < 0.05). This loss of brightness and darkening of the tissue are common observations in oil‐preserved seafood. Kilinc and Cakli (2005) and Gokoglu et al. (2009) noted similar declines in marinated fish stored in oil, attributing these changes to pigment migration and minor nonenzymatic browning. Despite the decline in visual scores over time, appearance remained remarkably stable and acceptable across both groups, shifting from an initial value of 4.29 in both Samples A and B to a final value of 3.75 by Month 11; this was likely due to the vacuum packaging limiting oxygen exposure and preventing severe surface discoloration.

#### 3.3.2 Odor, Greasiness, and Flavor

The sensory attributes of odor, greasiness, and flavor are critical indicators of the consumer's perception of freshness and quality in oil‐preserved marinades. In this study, both samples maintained acceptable scores (< 3.00) for these parameters throughout the 11 months of storage, though distinct differences were observed between the formulations (Table 2).

Odor scores remained relatively high and stable for the first 8 months, eventually settling at 3.75 for both groups at Month 11. Similarly, flavor scores for Sample A remained consistent at 4.29–3.25, whereas Sample B experienced a sharper decline, particularly at Month 3 (2.86). This temporary dip in Sample B's flavor and odor scores is likely linked to the higher salt (8%) and acidity (3.9%) levels, which may have initially created a sharp profile before reaching a sensory equilibrium during the maturation process. The overall maintenance of these scores is a direct result of the pre‐treatment boiling (100°C, 3 min) and microbial stasis, as no bacterial off‐odors were produced. Greasiness scores remained stable, beginning at 3.86 for both groups and ending at 3.25 for Sample A and 3.50 for Sample B. This suggests that the initial heat‐induced stabilization of the muscle matrix prevented excessive uptake of the sunflower oil, avoiding an unappealing, oily mouthfeel throughout the 11‐month storage.

#### 3.3.3 Texture and Overall Acceptability

Texture and overall acceptability scores for both groups remained above the limit of 3.00 until the end of the 11‐month storage period, confirming the long‐term success of the preservation method. Sample A maintained a more consistent profile, whereas Sample B exhibited a temporary decline at Month 3 (2.64) before recovering by Month 8. This fluctuation in Sample B likely reflects a “maturation phase” where the higher salt and acid levels were still equilibrating, as well as subjective rating differences among panel members and natural variations between individual specimens. Despite these minor shifts, the 11‐month shelf life achieved in this study significantly exceeds the 6–7 month limits typically reported for similar marinated products (Bilgin Fıçıcılar et al. 2018; Bilgin Fıçıcılar and Gençcelep 2020; Kilinc and Cakli 2005; Özden 2005), demonstrating the superior efficacy of the current formulation.

The gradual decline in sensory scores was consistent with the physicochemical changes observed during storage. Increases in FFA and TBA values indicate lipid hydrolysis and oxidation, which may influence flavor, odor, and greasiness, whereas increases in TVB‐N and pH reflect the accumulation of volatile nitrogenous compounds associated with quality deterioration. Nevertheless, these changes remained within acceptable limits, allowing the products to retain acceptable sensory quality throughout the storage period.

### 3.4 Color

The L* values of marinated *R. venosa* significantly decreased during storage (*p* < 0.05), indicating progressive darkening of the product (Table 3). Since rapana muscle naturally presents a pale color and low pigment content (Alkan and Alkan 2022), the reduction in lightness may be attributed to oxidative reactions occurring in the oil‐based marinade system, leading to protein–lipid interaction products during prolonged storage (Lund et al. 2011).

**TABLE 3:** Color parameters of marinated *Rapana venosa* during storage.

Parameter	Month	Sample	
		A	B
L*	0	63.78 ± 5.13^aA^	62.03 ± 4.83^aA^
	3	63.61 ± 1.13^aA^	62.75 ± 3.83^aA^
	8	58.76 ± 1.69^bA^	56.59 ± 1.34^bA^
	11	53.78 ± 2.35^cA^	53.41 ± 1.21^cA^
a*	0	−0.09 ± 0.57^aA^	0.52 ± 0.83^aA^
	3	−0.83 ± 1.03^bA^	0.13 ± 0.29^aA^
	8	−0.90 ± 0.69^bA^	−0.22 ± 0.70^bA^
	11	−1.28 ± 0.80^cA^	−0.90 ± 0.69^cA^
b*	0	10.23 ± 0.17^aA^	10.28 ± 3.94^aA^
	3	5.89 ± 3.93^bA^	9.91 ± 0.70^aA^
	8	11.78 ± 1.74^aA^	11.43 ± 1.51^aA^
	11	11.38 ± 1.41^aA^	12.14 ± 1.09^aA^

*Note:* Values are expressed as mean ± standard deviation (*n* = 3). Different lowercase letters indicate significant differences among storage times within the same formulation, whereas different uppercase letters indicate significant differences between formulations at the same storage time (*p* < 0.05). Sample A: 3.8% acetic acid, 5% salt. Sample B: 3.9% acetic acid, 8% salt.

The a* values shifted slightly toward negative values over time, suggesting a minor tendency toward greenish tones. However, these changes were limited in magnitude and may not be visually perceptible.

The b* values remained positive throughout storage, reflecting the preservation of yellowish characteristics, likely influenced by the sunflower oil matrix. Minor fluctuations were observed, particularly at Month 3 in group A.

No significant differences were observed between groups at the same storage period, indicating that storage time had a more pronounced effect on color changes than formulation differences.

### 3.5 Fatty Acid Compositions of Raw and Marinated *Rapana venosa* Samples

Table 4 presents the fatty acid composition of raw *R. venosa* meat and the two marinated samples (A and B). The data clearly demonstrate a substantial modification of lipid profile following marination. While raw rapana meat was characterized by a relatively balanced distribution of saturated (31.35%), monounsaturated (21.25%), and polyunsaturated fatty acids (47.40%), marinated samples exhibited a marked reduction in saturated fatty acids (SFA) and a pronounced increase in the monounsaturated fatty acids (MUFA) and polyunsaturated fatty acids (PUFA) fractions. In particular, linoleic acid (C18:2n6c) became the dominant fatty acid in marinated samples (> 55%), whereas long‐chain n‐3 polyunsaturated fatty acids such as eicosapentaenoic acid (EPA) and Docosahexaenoic acid (DHA), which were abundant in raw meat (6.07% and 19.26%, respectively), were almost completely absent after marination.

**TABLE 4:** Fatty acid composition (% of total fatty acids) of raw and marinated *Rapana venosa* samples.

Fatty acid	Raw rapana meat (%)	Marinated Sample A (%)	Marinated Sample B (%)
**Saturated fatty acids (SFA)**	31.35	12.03	12.09
C15:0 (pentadecanoic acid)	1.00	—	—
C16:0 (palmitic acid)	8.86	6.64	6.64
C17:0 (heptadecanoic acid)	3.35	—	—
C18:0 (stearic acid)	6.87	4.18	4.21
C20:0 (arachidic acid)	0.42	0.30	0.31
C22:0 (behenic acid)	0.29	0.83	0.85
C23:0 (tricosanoic acid)	8.52	—	—
**Monounsaturated fatty acids (MUFA**)	21.25	31.36	31.48
C16:1 (palmitoleic acid)	2.18	0.11	0.11
C18:1n9c (oleic acid)	5.18	31.07	31.19
C20:1 (eicosenoic acid)	8.57	—	—
C22:1 (erucic acid)	0.56	—	—
C24:1 (nervonic acid)	4.76	0.18	0.18
**Polyunsaturated fatty acids (PUFA)**	47.40	56.60	56.43
C18:2n6c (linoleic acid)	3.01	55.98	55.75
C18:3n3/n6 (linolenic acid)	0.90	0.24	0.15
C20:2 (eicosadienoic acid)	2.16	0.09	0.23
C20:3 (eicosatrienoic acid)	0.35	—	—
C20:4n6 (arachidonic acid)	10.08	—	—
C22:2 (docosadienoic acid)	5.57	—	—
C20:5n3 (EPA)	6.07	0.29	0.30
C22:6n3 (DHA)	19.26	—	—
∑n‐3	26.23	0.29	0.30
∑n‐6	13.09	56.22	55.90

*Note:* Sample A: 3.8% acetic acid, 5% salt. Sample B: 3.9% acetic acid, 8% salt.

The fatty acid profile of raw *R. venosa* meat shows a typical marine mollusk lipid composition rich in long‐chain n‐3 polyunsaturated fatty acids. Marine gastropods and bivalves are widely reported to contain significant amounts of EPA and DHA (Joseph 1982). The high DHA level observed in the present study (19.26%) is consistent with previous reports indicating that molluscan species can accumulate substantial amounts of n‐3 LC‐PUFAs due to dietary phytoplankton intake (Panayotova et al. 2019; Popova et al. 2017).

In contrast, both marinated samples (A and B) displayed a fatty acid profile dominated by linoleic acid (~56%) and oleic acid (~31%), which are characteristic components of sunflower oil (Aşkın 2018; Kucukboyaci et al. 2003). The disappearance of DHA and the drastic reduction of EPA (to ~0.3%) suggest a dilution effect caused by oil incorporation rather than selective oxidative degradation. Similar alterations in fatty acid composition following vegetable oil‐based marination have been reported in processed seafood products (Özden 2005).

## 4 Conclusion

In this study, *R. venosa* meat was marinated using two different formulations and stored at 4°C for 11 months. Microbiological, physicochemical, sensory, and fatty acid analyses demonstrated that both marinated products remained microbiologically safe and chemically stable throughout storage. No microbial growth was detected in either formulation during the entire storage period.

Although pH, FFA, TBA, and TVB‐N values increased significantly during storage, all parameters remained within acceptable quality limits. Sensory scores showed a gradual decline during storage. Although some individual sensory attributes fell below the acceptability threshold at specific sampling times, overall acceptability remained above 3.00 at Month 11 in both formulations. The formulation containing higher salt and acidity exhibited slightly better control of pH and TVB‐N accumulation, indicating enhanced inhibition of endogenous enzymatic activity.

These findings indicate that the observed physicochemical and sensory changes were associated with normal storage‐related deterioration processes rather than product spoilage. Despite gradual changes in quality attributes, all measured parameters remained within acceptable limits throughout the 11‐month refrigerated storage period, confirming the effectiveness of the preservation system.

Marination caused a pronounced modification of the fatty acid profile. While raw *R. venosa* meat was characterized by high levels of long‐chain n‐3 polyunsaturated fatty acids (particularly EPA and DHA), marinated samples were dominated by linoleic and oleic acids due to sunflower oil incorporation. The marked reduction of marine‐derived n‐3 fatty acids reflects dilution by the oil phase rather than selective degradation. This finding highlights that oil‐based marination significantly alters the nutritional lipid profile of the final product.

Overall, the marination process effectively inhibited microbial growth, delayed physicochemical deterioration, preserved acceptable sensory properties, and extended shelf life up to 11 months under refrigeration. Therefore, cold marination combined with oil preservation represents a safe and practical strategy for valorizing *R. venosa* as a ready‐to‐eat seafood product and supports the sustainable utilization of this economically important invasive species.

## Author Contributions

**Bilge Bilgin Fıçıcılar:** conceptualization, investigation, writing – original draft, formal analysis, supervision, visualization, methodology. **Koray Korkmaz:** funding acquisition, writing – review and editing, validation, software, data curation.

## Conflicts of Interest

The authors declare no conflicts of interest.

## Data Availability

The data that support the findings of this study are available from the corresponding author upon reasonable request.
